# The Impact of VCAM-1 expression on left ventricular performance following acute coronary syndromes

**DOI:** 10.25122/jml-2025-0083

**Published:** 2025-05

**Authors:** Ruxandra Copciag, Vladimir Bratu, Roxana Rimbas, Laura Lungeanu, Alexandru Corlan, Alexandru Schiopu, Maya Simionescu, Dragos Vinereanu

**Affiliations:** 1 Department of Cardiology and Cardiovascular Surgery, Bucharest Emergency University Hospital, Carol Davila University of Medicine and Pharmacy, Bucharest, Romania; 2 Bucharest Emergency University Hospital, Bucharest, Romania; 3 Skåne University Hospital Lund, Lund University, Malmö, Sweden; 4 Institute of Cellular Biology & Pathology Nicolae Simionescu, Bucharest, Romania

**Keywords:** 3D echocardiography, acute coronary syndrome, left ventricular remodeling, VCAM-1, 3-DE: 3-Dimensional Echocardiography, ACS: Acute Coronary Syndrome, AMI: Acute Myocardial Infarction, CMR: Cardiac Magnetic Resonance, CV: Cardiovascular, EF: Ejection Fraction, LVEDV: Left Ventricular End-Diastolic Volume, LVEF: Left Ventricular Ejection Fraction, LV: Left Ventricle, PCI: Percutaneous Coronary Intervention, SPSS: Statistical Package for the Social Sciences, STEMI: ST-Elevation Myocardial Infarction, Svcam-1: Soluble(Serum) Vascular Cell Adhesion Molecule-1, VCAM-1: Vascular Cell Adhesion Molecule-1

## Abstract

Inflammatory pathways, particularly those involving vascular cell adhesion molecule-1 (VCAM-1), play a central role in post-ischemic myocardial remodeling. However, its relationship with left ventricle (LV) performance or volumetric changes has not been systematically examined. This study aimed to investigate the association between circulating VCAM-1 levels, LV volumes, and LV ejection fraction (LVEF), quantified using three-dimensional echocardiography (3-DE), in patients with acute coronary syndrome (ACS). All patients underwent comprehensive clinical evaluation and 3-DE assessment within the first 24 hours of hospital admission. Concurrently, a full panel of locally available laboratory tests was performed, including serum sampling for VCAM-1 analysis. A follow-up evaluation, comprising repeated biological and echocardiographic measurements, was conducted two months after the index event. A total of 90 patients with ACS (mean age 54 ± 9 years; 75 males) were included in the analysis. Among these, 30 patients (33.3%) had a ≥10% increase in left ventricular end-diastolic volume (LVEDV) at follow-up, indicative of adverse left ventricular remodeling. Baseline VCAM-1 levels were significantly correlated with subsequent changes in LVEDV and LVEF from admission to follow-up (r = -0.42, *P* < 0.05, and r = -0.43, *P* < 0.05, respectively). Furthermore, the dynamic changes in VCAM-1 between assessments also showed significant correlations with changes in LVEDV and LVEF (r = 0.41, *P* < 0.05; r = -0.46, *P* < 0.05). This study suggests that VCAM-1, an inflammatory biomarker, may be a prognostic indicator of LV remodeling and dysfunction in patients with acute coronary syndromes. The findings support further exploration of VCAM-1 for risk stratification and therapy.

## INTRODUCTION

In recent years, numerous immunological biomarkers have been investigated for their potential association with cardiovascular diseases, as they may contribute to improved prognostic assessment. Among these, vascular cell adhesion molecule-1 (VCAM-1), a member of the immunoglobulin superfamily, has emerged as a key mediator in the inflammatory response. Its soluble form (sVCAM-1) is upregulated predominantly by pro-inflammatory cytokines in the vascular endothelium, where it facilitates the adhesion, recruitment, and transmigration of immune cells, including monocytes, lymphocytes, basophils, and eosinophils [1]. Elevated levels of sVCAM-1 have been consistently observed in patients with acute coronary syndromes (ACS) [2,3]. Importantly, sustained increases in sVCAM-1 concentrations have been linked to a higher incidence of major adverse cardiovascular events over 6 months [4]. Moreover, in patients with acute myocardial infarction (AMI), increased serum sVCAM-1 levels have shown a strong association with the later development of heart failure following ST-segment elevation, underscoring the biomarker’s prognostic relevance in this clinical setting [5]. Although the pro-inflammatory function of VCAM-1 in the pathogenesis of atherosclerosis and acute coronary syndromes is well-documented, efforts to translate this knowledge into targeted therapies have only recently developed. Emerging therapeutic approaches are increasingly directed toward the selective inhibition of VCAM-1 [6]. However, the association between VCAM-1 and echocardiographic parameters commonly used for risk stratification and prognostic assessment in patients with ACS remains insufficiently explored. To date, no studies have comprehensively evaluated its relationship with standard echocardiographic indices like left ventricular (LV) volumes and LV ejection fraction (LVEF).

Current echocardiographic guidelines for chamber quantification recommend three-dimensional (3D) imaging to evaluate LV volumes and ejection fraction. This approach offers enhanced accuracy in assessing LV morphology, demonstrates high reproducibility, and avoids the limitations associated with geometric assumptions [7]. Moreover, measurements derived from three-dimensional echocardiography (3-DE) have shown strong agreement with those obtained by cardiac magnetic resonance imaging (CMR), the current gold standard in LV assessment [8].

Thus, our main objective was to analyze the association between soluble VCAM-1(sVCAM-1) values, left ventricular end-diastolic volume (LVEDV), and LVEF assessed by 3-DE in patients with ACS.

## MATERIAL AND METHODS

### Study population and design

This was a prospective, single-center study that enrolled patients diagnosed with acute coronary syndrome, with or without ST-segment elevation, who underwent coronary angiography and received optimal medical treatment in accordance with current clinical guideline recommendations [9,10]. The study protocol was approved by the institutional ethics committee, and written informed consent was obtained from all participants prior to enrollment.

Inclusion criteria were as follows: (i) age ≥18 years; (ii) hospitalization for ACS confirmed according to guideline definitions [9,10]; and (iii) adequate acoustic windows allowing reliable echocardiographic imaging. Exclusion criteria included: (i) arrhythmias that precluded 3DE image acquisition; (ii) presence of significant valvular heart disease; (iii) hemodynamic instability; and (iv) pathologies leading to systemic inflammation and decreased life expectancy. Baseline data collection included patient demographics, clinical characteristics, laboratory parameters, three-dimensional echocardiographic assessment of left ventricular ejection fraction and LV volumes, angiographic findings, and pharmacological treatment. The initial evaluation was conducted within the first 24 hours following admission for acute coronary syndrome. A follow-up assessment, comprising the same clinical, laboratory, and echocardiographic parameters, was carried out 6 to 8 weeks after the baseline evaluation.

### Biological assessment

A comprehensive panel of laboratory tests was performed as part of the study protocol, encompassing routine hematological and biochemical analyses. These included complete blood count, erythrocyte sedimentation rate (ESR), coagulation profile, fasting plasma glucose, glycated hemoglobin (HbA1c), liver function tests, renal biomarkers (creatinine, urea, uric acid), serum electrolytes, lipid profile (total cholesterol, high-density lipoprotein [HDL], low-density lipoprotein [LDL], and triglycerides) and high-sensitivity cardiac troponin I (Pathfast; LSI Medience). Serum concentrations of soluble VCAM-1 (sVCAM-1) were measured using a commercially available sandwich ELISA kit (Quantikine Human VCAM-1/CD106 ELISA, R&D Systems, Minneapolis, MN, USA), according to the manufacturer’s instructions. The same blood samples were obtained at follow-up.

### Echocardiography

**Figure 1:**
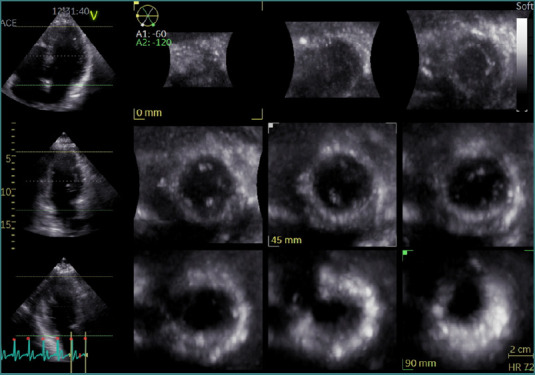
Acquiring a three-dimensional (3D) echocardiographic image of the left ventricle using a multi-beat full-volume technique

**Figure 2:**
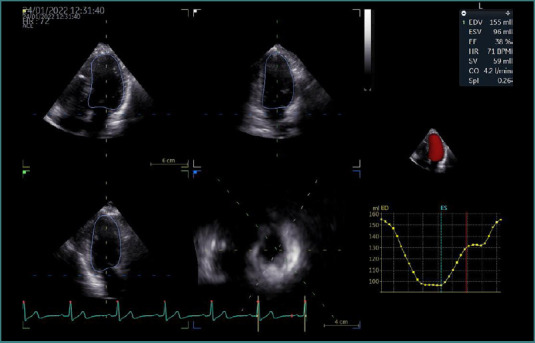
Left ventricular volume measurements and LV ejection fraction calculation, using the dedicated software. The 3D shape of LV and the volumetric curve were automatically obtained after tracing the endocardial border of the LV.

A comprehensive transthoracic echocardiographic examination was conducted in accordance with current guideline recommendations [11], utilizing advanced imaging systems (Vivid E9 or Vivid E95, GE Healthcare). Image acquisition employed an M5S transducer for two-dimensional echocardiography (2DE) and a 4V transducer for 3DE. All echocardiographic data were obtained within 24 hours of hospital admission. For 3D volumetric analysis of the left ventricle, full-volume datasets were acquired from the apical four-chamber view using a multibeat approach. This method captures multiple sub-volumes over consecutive cardiac cycles, which are then stitched together to form a comprehensive 3D representation of the LV. This approach enhances spatial resolution and provides a more accurate assessment of LV volumes and ejection fraction compared to two-dimensional echocardiography. However, it requires the patient to maintain a breath-hold and a regular heart rhythm during acquisition to minimize stitching artifacts (Figure 1). Offline analysis was performed using the EchoPAC software suite (version 203, GE Healthcare), and left ventricular volumes and ejection fraction were calculated via the 4D Auto LVQ tool (GE Vingmed Ultrasound), as illustrated in Figure 2.

### Statistical analysis

Statistical analysis was conducted using SPSS software, version 25.0 (IBM Corp., Armonk, NY, USA). Continuous variables were summarized as mean ± standard deviation (SD), while categorical data were reported as absolute numbers and corresponding percentages. Comparisons between categorical variables were carried out using the chi-squared (χ^2^) test. Pearson’s correlation coefficient was applied to assess linear associations between continuous variables. A correlation coefficient (r) greater than 0.40 was interpreted as indicative of a moderate to strong relationship, and statistical significance was defined as a *P* value less than 0.05.

## RESULTS

### Study population

Data from 90 patients (54 ± 9 years, 75 men) admitted with ACS (21.1% with non-ST elevation ACS and 78.9% with ST-elevation ACS) were analyzed. Table 1 presents an overview of patient characteristics, including comorbid conditions and angiographic findings. The clinical parameters shown in Table 1 were obtained before transthoracic echocardiography (TTE). The mean sVCAM-1 value assessed within the first 24 hours of admission was 4,65 ± 0.33, while the sVCAM-1 value at follow-up was 4,72 ± 0.29 (*P* = 0.04). Mean 3-DE LVEF at baseline was 47 ± 8%. All participants underwent coronary angiography and received evidence-based medical therapy following current clinical guidelines [12,13]. (Table 2). All patients were treated with acetylsalicylic acid and a high-intensity statin regimen. Additional pharmacologic therapies were prescribed based on individual clinical indications and patient tolerance.

**Table 1:** Characteristics of the study population

Clinical characteristics	All patients (*n* = 90)
Age, years	54 ÷ 9
Sex, male	75 (83.3)
BMI > 25 kg/m^2^	76 (84.4)
Hypertension	59 (65.6)
Diabetes	23 (25.6)
Dyslipidemia	52 (92.8)
LDL-c > 100 mg/dI	74 (82.2)
Smoking	61 (67.8)
PAD	2 (2.2)
CKD	41 (45.6)
AMI type - ST- segment elevation	71 (78.9)
One-vessel disease	53 (58.9)
Two-vessel disease	27 (30.0)
Three-vessel disease	10 (11.1)
Symptom-to-ballon time (h)	6.3 [1-24]
CRP within 24 hours (lab range 1-5 mg/dl)	23.53 [1-290]
hs-cTn I at admission (ug/L)	11156,11 [40-50000]
WBC (10^9^/L)	11.4 [5.6-20.5]
Baseline 3-DE LVEF (%)	47 ± 8
3-DE LVEDV increase from baseline to follow-up	30 (33)

Data are presented as mean ± SD, median [interquartile range], or n (%) 3-DE, 3D echocardiography; AMI, acute myocardial infarction; BMI, body mass index; CKD, chronic kidney disease; CRP, C-reactive protein; LVEDV, left ventricular end-diastolic volume; LVEF, left ventricular ejection fraction; PAD, peripheral artery disease; WBC, white blood cells.

**Table 2:** Pharmacologic therapies received during hospitalization and at discharge, respectively

Pharmacotherapy	All patients (*n* = 90)
Thrombolysis	16 (17.8)
Aspirin	100 (100)
Ticagrelor	83 (92.2)
Clopidogrel	6 (6.7)
Beta-blocker	85 (94.4)
Angiotensin converting enzyme inhibitor/ angiotensin receptor blocker	68 (75.6)
Statin	90 (100)
Mineralocorticoid receptor antagonist	24 (26.7)

Data are presented in n (%)

### Correlation between sVCAM-1 at baseline and changes in 3-DE LVEDV and LVEF

**Figure 3:**
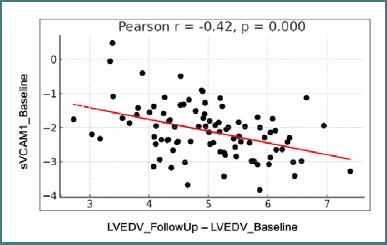
Correlation between sVCAM1 values at baseline and changes in LVEDV between visits LVEDV - left ventricular end-diastolic volume

There was a significant negative correlation between sVCAM-1 levels at baseline and the change in end-diastolic volume between baseline and follow-up (r = -0.42, *P* < 0.05; Figure 3). A negative correlation means that higher VCAM-1 levels at baseline are associated with a greater increase in LV volume over time.

**Figure 4:**
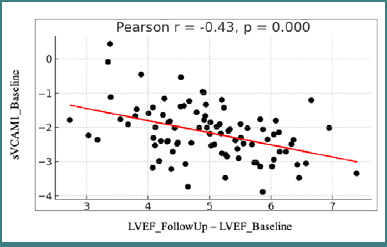
Correlation between sVCAM1 values at baseline and changes in LVEF between visits LVEF - left ventricular ejection fraction

Baseline VCAM-1 levels were also negatively correlated with the change in LVEF between the two assessments, at admission and after 6-8 weeks, respectively (r = –0.43, *P* < 0.05), indicating that higher VCAM-1 concentrations at admission were associated with a reduction in 3-DE LVEF at follow-up (Figure 4).

### Correlation between changes in VCAM-1 from baseline to follow-up and changes in 3-DE LVEDV and LVEF

**Figure 5:**
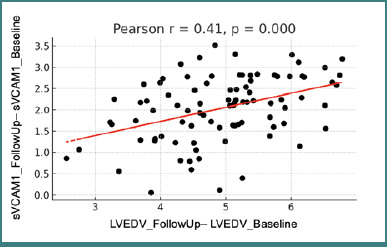
Correlation between changes in sVCAM1 values and changes in LVEDV between visits LVEDV - left ventricular end-diastolic volume

A statistically significant positive correlation was observed between the change in three-dimensional left ventricular end-diastolic volume and VCAM-1 levels from baseline to follow-up (r = 0.41, *P* < 0.05). Patients who exhibited a greater increase in VCAM-1 concentrations over time also tended to demonstrate a more pronounced increase in LV end-diastolic volume as measured by 3-DE (Figure 5).

**Figure 6:**
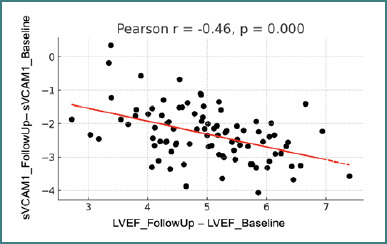
Correlation between changes in sVCAM1 values and changes in LVEF between visits LVEF - left ventricular ejection fraction

There was also a statistically significant negative correlation between the changes in LVEF and the changes in VCAM-1 levels over time (r = -0.46, *P* < 0.05; Figure 6). Patients who experienced a greater increase in VCAM-1 concentrations between baseline and follow-up tended to show a greater decline in LVEF.

## DISCUSSION

In the present study, patients with ACS who exhibited higher VCAM-1 levels at admission demonstrated a greater increase in LV end-diastolic volume assessed by 3-DE at follow-up, 6 to 8 weeks post-event, suggesting a tendency toward adverse ventricular remodeling. Our findings also indicate that higher baseline levels of VCAM-1 were associated with a decline in LVEF over the follow-up period. This inverse relationship suggests that an elevated inflammatory state during the acute coronary event may contribute to progressive deterioration of LV performance. To our knowledge, this is the first study assessing the relationship between sVCAM-1 levels and LV performance assessed by 3-DE. These results reinforce the potential role of VCAM-1 as a marker of adverse ventricular remodeling and functional decline in the subacute phase following myocardial infarction. However, previous data demonstrated that VCAM-1 serum levels assessed in patients with myocardial infarction who were brought to the emergency department were excellent indicators of heart failure after ACS. Specifically, they were considerably higher in patients with post-ST-segment elevation who developed symptoms of heart failure at discharge [1,14,15]. In a study involving 75 patients with acute coronary syndrome, researchers assessed circulating levels of VCAM-1. The findings indicated that elevated VCAM-1 levels were a strong predictor of major adverse cardiovascular events, including new coronary incidents, hospitalizations for angina, and cardiac death. Postadzhiyan *et al*. [16] suggested that cell adhesion molecules like VCAM-1 might serve as more effective prognostic markers than traditional inflammatory indicators like high-sensitivity C-reactive protein in predicting cardiovascular events among ACS patients.

In addition to evaluating baseline sVCAM-1 levels, our study also examined their serum dynamics two months following the ACS event. An increase in VCAM-1 levels from baseline to follow-up was significantly associated with markers of adverse ventricular remodeling, including both an increase in left ventricular end-diastolic volume (as assessed by 3D echocardiography) and a decline in LVEF, reflecting impaired systolic function. These findings suggest that a progressive rise in VCAM-1, indicative of ongoing inflammatory activation, may play a key role in the structural and functional deterioration of the left ventricle following acute coronary syndrome. These findings align with previous data showing that an increase in sVCAM-1 over time after an ACS is associated with poor clinical outcomes. In a prospective study enrolling more than two hundred patients with STEMI, in which sVCAM-1 serum levels were collected at admission, 4, 24, 48 h, and 1 month after admission, Hayek *et al*. demonstrated that there is a sustained endothelial activation evaluated by sVCAM-1 release. sVCAM-1 serum levels increase within the first month following STEMI and are associated with infarct size, LV remodeling, and adverse clinical events at one year. These observations are of major clinical relevance as they warrant consideration around sVCAM-1 as an early biomarker of poor prognosis in STEMI patients [17]. The evolution of serum sVCAM-1 levels after an ACS suggests that inflammation persists beyond the acute phase of the event, since previous data show a significant increase at three months compared to values at the time of presentation. The continued elevation of sVCAM-1 in patients with unstable angina or non–Q-wave myocardial infarction indicates a sustained inflammatory response lasting up to six months, likely reflecting ongoing immune cell activity at sites of vascular injury [18].

Given the growing recognition of left ventricular remodeling as a critical determinant of post-ischemic outcomes, exploring the role of VCAM-1 in this process is of considerable clinical interest. While the pro-inflammatory function of VCAM-1 in the development of atherosclerosis is well documented, efforts to translate this understanding into targeted therapies have only recently accelerated. Emerging therapeutic strategies increasingly focus on disrupting the interaction between VCAM-1 and very late antigen-4 (VLA-4), a key pathway mediating monocyte adhesion to the vascular endothelium. Among these, peptide-based and monoclonal antibody-based approaches have shown particular promise. Although current challenges, such as delivery mechanisms and translational feasibility, persist, ongoing advances in molecular engineering and drug delivery technologies pave the way for more effective and clinically viable interventions [6].

### Study limitations

This study has several limitations that should be acknowledged. First, the relatively small sample size may limit the statistical power. Additionally, the cohort included patients with varying clinical presentations and angiographic profiles, which may introduce heterogeneity in the observed outcomes. The follow-up period was relatively short, capturing early changes in ventricular remodeling but potentially missing longer-term structural and functional alterations. As such, while our findings suggest a potential link between sVCAM-1 levels and left ventricular remodeling, larger studies with longer follow-up durations are necessary to validate these observations and to establish a more definitive relationship between VCAM-expression, adverse remodeling, and the progression to heart failure.

## CONCLUSION

This study highlights the potential role of VCAM-1, a biomarker of inflammation and immune activation, as a prognostic indicator of impaired LV performance, as reflected by changes in left ventricular ejection fraction and left ventricular end-diastolic volume, following acute coronary syndromes. These findings underscore the importance of inflammatory pathways in cardiac remodeling and support further investigation of VCAM-1 in risk stratification and therapeutic targeting in patients with ACS.

## Data Availability

Data are contained within this article.
